# Alectinib combined with VRCD and BV monoclonal antibody for the treatment of ALK-positive large B-cell lymphoma: a case report and literature review

**DOI:** 10.3389/fonc.2024.1480880

**Published:** 2025-01-21

**Authors:** Hongxia Wang, Junjun Bai, Bei Zhang, Zhixin Pei, Yingxin Zhao, Jingjing Gu, Zhiyu Fang, Miaomiao Liu, Xun Liang, Qinglin Song

**Affiliations:** ^1^ Department of Hematology, Jiaozuo People’s Hospital, Jiaozuo, Henan, China; ^2^ College of Resources and Environment, Henan Polytechnic University, Jiaozuo, Henan, China

**Keywords:** ALK+ LBCL, alectinib, bortezomib, lenalidomide, BV monoclonal antibody

## Abstract

We report a case of a 53-year-old male patient who was diagnosed with anaplastic lymphoma kinase-positive large B-cell lymphoma. A pathological examination showed plasma-cell immunophenotype negative for mature B-lymphocyte markers. The patient received three cycles of an “alectinib + Vincristine + Rituximab + Cyclophosphamide + Doxorubicin (VRCD) “ regimen. In the fourth cycle, brentuximab vedotin monoclonal antibody treatment was added to increase the efficacy of the treatment. Positron emission tomography-computed tomography and a bone marrow examination were carried out after four cycles, revealing a complete response. Stem cells were successfully harvested. Thus far, the patient has undergone six treatment cycles and successfully received autologous hematopoietic stem cell transplantation. Currently, the patient is receiving maintenance therapy with alectinib and lenalidomide and remains in a favorable clinical condition.

## Introduction

Anaplastic lymphoma kinase-positive large B-cell lymphoma (ALK+ LBCL) is a rare invasive B-cell lymphoma that has plasmablast/immunoblast morphology and ALK fusion protein expression (1, 2). It has a poor prognosis (with a median survival of 1.8 years) and low sensitivity to conventional systemic chemotherapy (3). There are reports of encouraging efficacy of a myeloma-like regimen in patients with ALK+ LBCL. Here, we present a case of a patient treated with the ALK inhibitor alectinib in combination with the VRCD chemotherapy regimen, supplemented with the addition of brentuximab vedotin (BV) monoclonal antibody treatment during maintenance therapy. The patient achieved a satisfactory response.

## Case report

**Figure 1:**
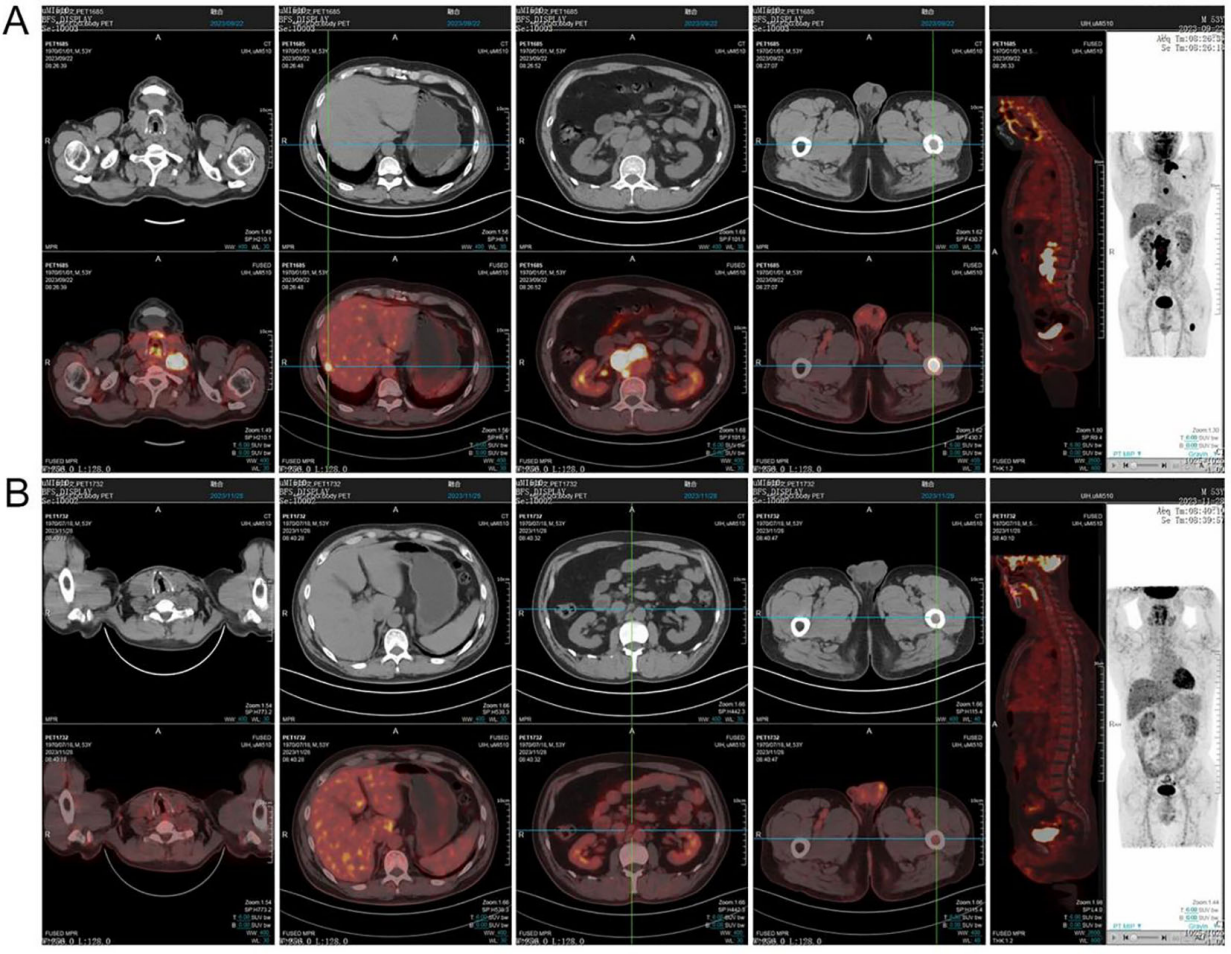
PET-CT imaging was performed at the time of initial diagnosis **(A)** and after two cycles of chemotherapy **(B)**.

**Figure 2:**
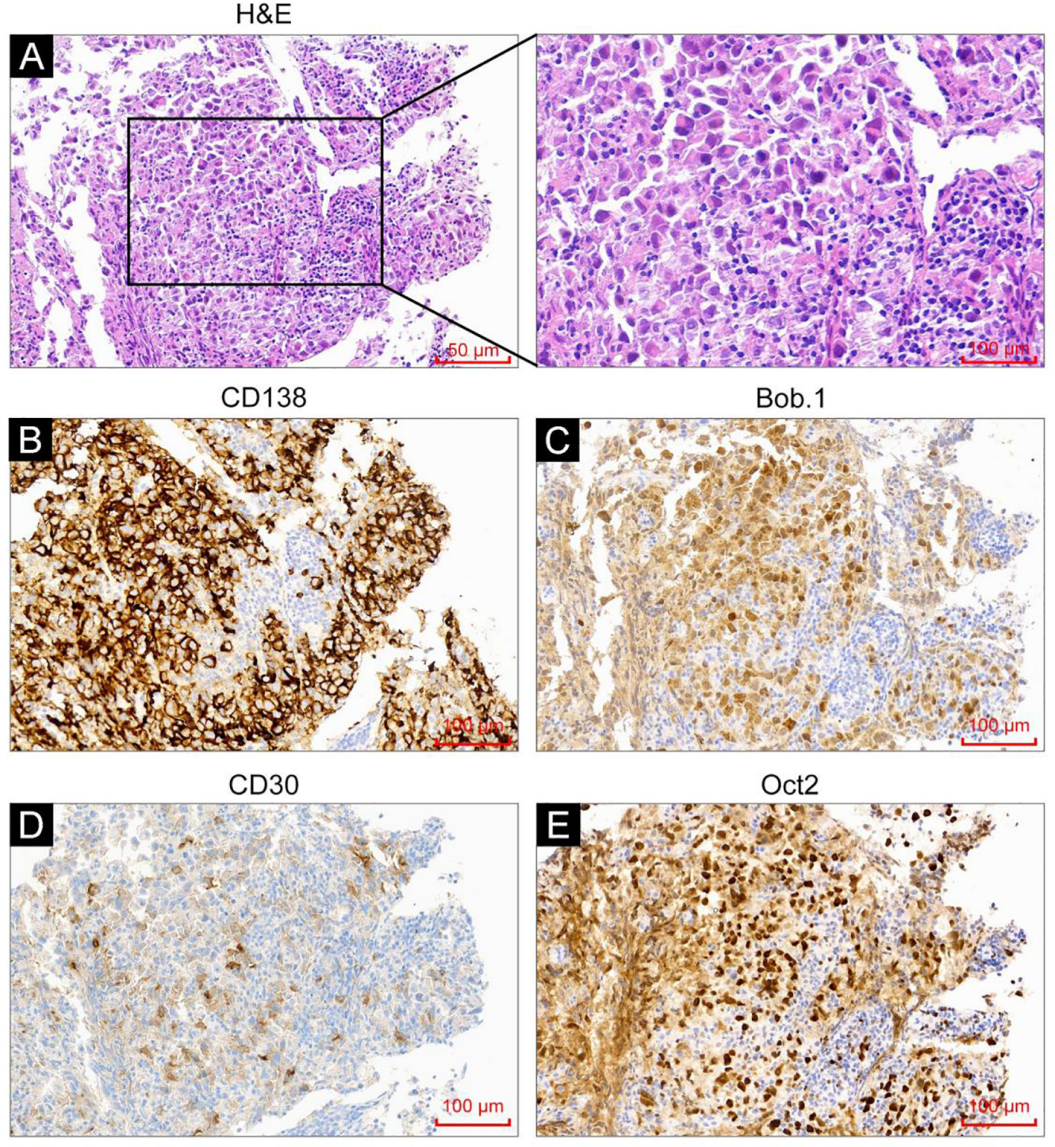
H&E staining and immunohistochemistry analysis were performed on the tumor tissues. **(A)** H&E staining showed tumor cells with immunoblast/plasma-cell morphology. **(B–E)** Immunohistochemistry analysis revealed CD138 positivity **(B)**, weak CD30 positivity **(C)**, Bob.1 positivity **(D)**, and Oct-2 positivity **(E)**.

In September 2023, a 53-year-old man was admitted due to fever, cough, and asthenia. Physical examination on admission showed multiple enlarged lymph nodes in the neck, axilla, and groin, with partial fusion and no tenderness. In the auxiliary examinations, a routine blood test showed a white blood cell count of 6.01×10^9^/L, red blood cell count of 4.53×10^12^/L, hemoglobin concentration of 137 g/L, and platelet count of 213×10^9^/L; and the lactate dehydrogenase test result was 296 U/L. No abnormal lymphocytes were seen in bone marrow aspiration and bone marrow immunotyping examination. The karyotype in the bone marrow was 46, XY (5). Positron emission tomography-computed tomography (PET-CT) showed multiple soft-tissue nodular shadows in the left supraclavicular and subclavian regions, with partial fusion into clumps (**Figure 1A**). The largest clump had a maximum cross-sectional area of 37 mm × 22 mm and an SUVmax of 41.24. A soft-tissue nodule shadow was observed in mediastinum zone 7, with a long diameter of approximately 15 mm and an SUVmax of 36.03. A patchy slightly low-density shadow was seen in the right liver lobe, with blurred borders, a long diameter of approximately 13 mm and an SUVmax of 11.68. Multiple soft-tissue nodular shadows were found around the abdominal aorta and in the retroperitoneal region, with partial fusion into clumps. The largest clump had a maximum cross-sectional area of 34 mm × 20 mm and an SUVmax of 43.97. There was locally concentrated radioactivity in the left third and sixth ribs and right iliac bone, with an SUVmax of 12.59. A local density increase was found in the medullary cavity of the upper segment of the left femur, and the radioactivity was highly concentrated. The patient’s mediastinal blood pool SUVmax was 2.58 and liver blood pool SUVmax was 4.52. The pathological examination of the left cervical lymph node biopsy indicated ALK+ LBCL, namely lymphoid tissue hyperplasia, patchy diffuse infiltration, and the proliferation of morphologically abnormal cells with large volume, abundant cytoplasm, round or irregular nuclei, thin chromatin, and visible nucleoli (**Figure 2A**). Immunohistochemistry revealed the following findings (**Figures 2B–E**): ALK (90% +, cytoplasm +), CD3 (-), CD4 (+), CD5 (-), CD10 (NS), CD20 (-), CD79a (-),CK7 (-), Ki67 (80% +), LCA (weakly +), MUC5AC (-), S100 (-), Villin (-), Vim (-), EMA (partially +), TIA1 (-), GramB (-), CD138 (90% +), CD43 (-), CD30 (25% +), MUM1 (+), CD38 (-), CD163 (-), PAX5 (weakly +), Oct2 (+), kappa (-), lambda (+), Bob.1 (+), Vs38c (+), CD4 (-), IgA (low +), and EREB (-). Lymphoma next-generation sequencing showed positivity for CCND3 and CDK6. Tumor transcriptome sequencing (RNA-seq) identified a CLTC-ALK fusion mutation. The final diagnosis was ALK+ LBCL (stage IV, invading the left supraclavicular, subclavian, mediastinal, para-aortic, and retroperitoneal lymph nodes; right liver lobe; left third and sixth ribs; right iliac bone; and upper segment of the left femur). The patient underwent two chemotherapy cycles of an “alectinib + VRCD” regimen on 29 September and 27 October 2023 (alectinib 600 mg bid; bortezomib 1.3 mg/m^2^ on days 1, 4, 8, and 11; lenalidomide 25 mg/day, on days 1-14; cyclophosphamide 300 mg/m^2^ on days 1 and 8; dexamethasone 20 mg on days 1, 2, 4, 5, 8, 9, 11, and 12). A complete response (CR) was achieved on PET-CT evaluation (**Figure 1B**). On 30 November 2023, the “alectinib + VRCD” regimen was used for the third chemotherapy cycle. As the patient’s pathological examination showed CD30 (25% +), an “alectinib + VRCD + BV monoclonal antibody” regimen was used for the fourth cycle. After the four cycles, bone marrow analysis and PET-CT showed a CR and stem cells were successfully harvested (2.6×10^6^/L). After that, the patient underwent another two cycles of alectinib + VRCD. Throughout the patient’s treatment course, the only adverse event observed was alectinib-induced bradycardia, which was not accompanied by syncope or palpitations and did not require clinical intervention. In July 2024, the patient underwent autologous hematopoietic stem cell transplantation with a “BEAM” conditioning regimen, consisting of a simustine capsule (250 mg/m², qn, day -7), etoposide (200 mg/m², qd, days -6 to -3), cytarabine (200 mg/m², q12h, days -6 to -3), and melphalan (140 mg/m², qd, day -2). The patient is currently on maintenance therapy with alectinib and lenalidomide and remains in a state of sustained remission.

## Discussion

ALK+ LBCL is a type of rare CD20-negative invasive non-Hodgkin lymphoma (4), accounting for less than 1% of diffuse large B-cell lymphoma (DLBCL) cases. Thus far, fewer than 200 cases have been reported worldwide. ALK+ LBCL was first recognized in 1977. In the 2017 World Health Organization Classification of Tumors of Hematopoietic and Lymphoid Tissues, ALK+ LBCL was defined as an ALK-positive monomorphic large immunoblast-like B-cell invasive tumor with a plasma-cell phenotype. ALK+ LBCL can occur at any age, with a median age at onset of 38 years. The male-to-female ratio is approximately 3.5:1. The clinical presentation of patients with ALK+ LBCL is similar to that of DLBCL, and includes painless lymph node enlargement on onset, rapid progression, systemic lymph node involvement, and extranodal involvement, and involvement of soft tissues, nasopharynx, tonsils, thoracic wall, mediastinum, pulmonary hilum, liver, and spleen. Bone marrow and cerebrospinal fluid involvement are rare (5, 6).

ALK+ LBCL is mainly diagnosed pathologically. Specifically, it shows a solitary large immunoblast-like diffuse/intrasinusoidal growth pattern, round nuclei, abundant basophilic or bichromatic cytoplasm, and central nucleoli. The immunophenotype of tumor cells includes strong ALK expression (ALK gene rearrangement) and expression of plasma-cell markers CD38, CD138, and VS38C. CD20a, Pax-5, CD79, and B-cell markers are generally not expressed but B cell-specific transcription factors such as Bob.1 and Oct-2 are expressed. CD30 is usually negative, but some patients have weakly positive lesions (7). Regarding genetics, most patients show IgH gene clonal rearrangement, and the most common rearrangement is t (2; 17)(p23; q23), which expresses the CTL-ALK fusion protein (localized to the cytoplasm). Few patients have t (2; 5)(p23; q35) rearrangement, and express the NMP-ALK fusion protein (localized to the cytoplasm and nucleus) (6), and there is no MYC rearrangement.

As there is no standardized treatment for ALK+ LBCL, most patients are treated using B-cell lymphoma regimens. However, ALK+ LBCL responds poorly (8) to conventional chemotherapy such as cyclophosphamide, doxorubicin, vincristine, and prednisone (CHOP), or a CHOP-like regimen, and the median survival is 12-18 months. Therefore, it is important to examine the potential uses of new drugs, particularly ALK inhibitors. ALK inhibitors are small-molecule tyrosine kinase inhibitors that target ALK, MET, and ROS1. According to previous reports, ALK inhibitors are effective against anaplastic large cell lymphoma (ALCL), ALK-positive non-small cell lung cancer (NSCLC), and ALK+ LBCL (9–11). Crizotinib is a first-generation ALK inhibitor that has been proven effective against ALK+ LBCL but its duration of response is short. Wass et al. (12) reported a stage IV ALK+ LBCL patient who relapsed after six cycles of standard chemotherapy and autologous hematopoietic stem cell transplantation. The patient then received crizotinib and ultimately achieved a partial response (PR). However, the patient again experienced recurrence. A recent study has shown that potent ALK inhibitors (alectinib and lorlatinib) have good efficacy. Four relapsed/refractory (R/R) ALK+ LBCL patients (75% progressed after crizotinib treatment) received 600 mg alectinib twice daily for treatment; three of them achieved a CR and one achieved a PR. Two of these patients underwent allogeneic hematopoietic stem cell transplantation, and the other two patients experienced progression of disease (PD). Among the patients with PD, one achieved a CR after receiving 100 mg of lorlatinib per day (11). Takiar et al. (13) reported two CHOP-resistant ALK+ LBCL patients. One received crizotinib and achieved a good response, with a CR lasting for 6 years. Another patient initially achieved remission after crizotinib treatment, but experienced PD after 3 months. The patient was then switched to the second-generation ALK inhibitor alectinib, which was well-tolerated, but the response lasted for 6 months. After progression, the patient received third-generation lorlatinib and achieved a CR.

ALK+ LBCL often presents with plasma cell morphology and immunophenotype (positivity for CD38 and CD138). Chemotherapy regimens for plasma-cell tumors, such as proteasome inhibitors and immunomodulators, may be effective against ALK+ LBCL. Plasmablastic lymphoma (PBL) is another type of invasive CD20-negative DLBCL with plasma cell differentiation. A case report and case series showed that bortezomib alone or in combination therapy increased the response of PBL patients, particularly bortezomib combined with dose-adjusted etoposide, prednisone, vincristine, cyclophosphamide, and doxorubicin (V-EPOCH) (14). A study included 16 patients with PBL who received four cycles of the V-EPOCH regimen. Fifteen patients (94%) achieved a complete response, and the 5-year overall survival rate was 63% (15). A patient with PBL achieved a 12-month CR after receiving lenalidomide combined with bortezomib as salvage therapy (16). Li et al. reported a case of a patient with ALK+ LBCL who received bortezomib, lenalidomide, and dexamethasone (VRD) combined with an ALK inhibitor and achieved a CR. As hematopoietic stem cell transplantation could not be performed, lorlatinib was used for maintenance therapy, and the CR was maintained for more than 6 months (17). Based on the aforementioned literature analysis and the patient’s financial status, we selected the second-generation ALK inhibitor alectinib combined with a myeloma-like VRCD regimen for treatment and achieved a CR after two cycles.

BV is an antibody-drug conjugate that is synthesized by protease-sensitive cross-linking between the CD30-targeting monoclonal antibody and microtubule disruptor monomethyl auristatin E (MMAE). BV is stable in the blood and releases MMAE after it is endocytosed into CD30-expressing cells to kill target cells. CD30 expression is variable in DLBCL. A study on BV treatment of relapsed/refractory CD30-positive DLBCL reported a response rate of 44% (18). However, there are limited data on BV treatment in ALK-negative LBCL. A previous study reported a 23-year-old female patient with ALK+ LBCL who was misdiagnosed with anaplastic large cell lymphoma due to a diffuse expression of CD30. The patient received a pediatric ALCL99 regimen for treatment and progressed after two chemotherapy cycles. Considering the expression of CD30, BV was used for the treatment. Although she experienced a decrease in transient lactate dehydrogenase (LDH) levels, she progressed after two cycles and died 4 months after the initial diagnosis (19). After that, her specimen was reviewed, showing that the tumor originated from B cells, which was consistent with ALK+ LBCL. Our patient had 25% CD30 expression. Before stem cell harvesting, we added BV monoclonal antibody treatment, hoping to achieve further residue clearance. The patient maintained a CR and stem cells were harvested. Another cycle of BV monoclonal antibody consolidation treatment was planned before or after stem cell transplantation. Adding BV monoclonal antibody to the treatment of CD30+ ALK+ LBCL patients may increase treatment efficacy, but more samples are needed for further validation.

## Conclusion

Patients with ALK+ LBCL are not sensitive to chemotherapy and have a poor prognosis. An ALK inhibitor combined with a myeloma-like regimen is effective in these patients. In the targeted therapy era, different targeted therapy drugs combined with chemotherapy may improve prognosis in such rare lymphomas.

## Data Availability

The datasets of this study are available from the corresponding author on reasonable request.
